# A computational model of pattern separation efficiency in the dentate gyrus with implications in schizophrenia

**DOI:** 10.3389/fnsys.2015.00042

**Published:** 2015-03-25

**Authors:** Faramarz Faghihi, Ahmed A. Moustafa

**Affiliations:** ^1^Center for Neural Informatics, Structures, and Plasticity, Krasnow Institute for Advanced Study, George Mason UniversityFairfax, VA, USA; ^2^Department of Veterans Affairs, VA New Jersey Health Care SystemEast Orange, NJ, USA; ^3^School of Social Sciences and Psychology and Marcs Institute for Brain and Behaviour, University of Western Sydney SydneyNSW, Australia

**Keywords:** pattern separation, sparse spiking, sparse coding, feedback inhibition, connectivity rate, mutual information, dentate gyrus, entorhinal cortex

## Abstract

Information processing in the hippocampus begins by transferring spiking activity of the entorhinal cortex (EC) into the dentate gyrus (DG). Activity pattern in the EC is separated by the DG such that it plays an important role in hippocampal functions including memory. The structural and physiological parameters of these neural networks enable the hippocampus to be efficient in encoding a large number of inputs that animals receive and process in their life time. The neural encoding capacity of the DG depends on its single neurons encoding and pattern separation efficiency. In this study, encoding by the DG is modeled such that single neurons and pattern separation efficiency are measured using simulations of different parameter values. For this purpose, a probabilistic model of single neurons efficiency is presented to study the role of structural and physiological parameters. Known neurons number of the EC and the DG is used to construct a neural network by electrophysiological features of granule cells of the DG. Separated inputs as activated neurons in the EC with different firing probabilities are presented into the DG. For different connectivity rates between the EC and DG, pattern separation efficiency of the DG is measured. The results show that in the absence of feedback inhibition on the DG neurons, the DG demonstrates low separation efficiency and high firing frequency. Feedback inhibition can increase separation efficiency while resulting in very low single neuron’s encoding efficiency in the DG and very low firing frequency of neurons in the DG (sparse spiking). This work presents a mechanistic explanation for experimental observations in the hippocampus, in combination with theoretical measures. Moreover, the model predicts a critical role for impaired inhibitory neurons in schizophrenia where deficiency in pattern separation of the DG has been observed.

## Introduction

The brain’s ability to generate a representation of stimuli in the environment, as induced changes in neurons and neuronal networks, is essential for efficient memory and learning capabilities. The fundamental structure and function of many parts of the invertebrate and vertebrate brains are relatively well-studied. Neurons show rich morphological and electrophysiological variations in different brain regions and sub-regions. Different morphological and electrophysiological features of neurons are linked to dissociable neural functions (Barres et al., 1988; Zawadzki et al., 2012).

Moreover, the morphological and physiological properties of many kinds of neurons have been previously described. Exploring neural circuits and mechanisms of information processing that enables animals to live in dynamical environment play an important role in modern neuroscience (Clayton and Hen, 2005; Wilbrecht and Shohamy, 2009). Understanding the principal parameters which are involved in efficient stimuli encoding by biological neural systems plays an important role for understanding the neural mechanism of learning and memory, and designing bio-inspired systems that can perform complicated tasks (Yue et al., 2006; Rozo et al., 2013). Modeling and simulation studies aim to design bio-inspired neural systems (e.g., advanced robotic systems) that are capable of sensing stimuli and navigating in different environments such that they can perform some complicated tasks including human-like learning and memory processes (Stramandinoli et al., 2012). These efforts have led to the development of new generation of robots that can make decisions based on olfactory or auditory cues (Marjovi and Marques, 2011; Song et al., 2011).

Many parameters enable efficient information processing, including connectivity rate, firing threshold, and feedback inhibition among neurons. For example, morphological variations of neurons lead to different connectivity rate between different neurons and neural layers in the animal brain because it determines the flow of information that a neuron receives from its neighbors (Lamprecht and LeDoux, 2004; Seung and Sümbül, 2014). Single neurons as information processing units within neural population show a dynamic and activity-dependent synaptic strength. As an example, in trace eye-blink conditioning in mice, the contribution of hippocampal synaptic contact takes place at different moments during associative learning (Gruart et al., 2014). In some neurological disorders (e.g., Alzheimer’s disorder), a change in neural connectivity is associated with abnormalities in cognitive capabilities (He et al., 2009; D’Amelio and Rossini, 2012). The potential connectivity of neurons and their electrophysiological properties have been broadly studied in the rodent hippocampus (see: hippocampome.org/ for more information about comparing electrophysiological features of 122 neuron types in different sub-regions of hippocampus).

In addition to connectivity rate, variations in electrophysiological properties of a neuron affect its firing response to its input. One key electrophysiological feature is ‘firing threshold’ of a neuron because each neuron temporally integrates its inputs to firing threshold which, when met, results in spike trains (Ebner and Hameroff, 2011; Gupta et al., 2012). Although neurons can affect activity of other neurons by diffusing different chemicals (retrograde signals), spiking is believed to be the major method for neural communication. Another known parameter which is involved in efficient neural encoding is the balance between inhibition that a neuron receives in the networks and excitatory inputs of neurons. This balance plays a critical role in neural information processing (Sengupta et al., 2013). Recently, the role of the balance of excitation and inhibition among neurons in hippocampus functionality has been shown (English et al., 2014). Inhibitory neurons are found in all parts of the animal brain such that any impairment in their function is associated with psychiatric and neurological disorders (Konradi et al., 2011). The role of the balance between excitation and inhibition has been shown using simulation studies to be critical in keeping the efficiency of neurons in response to presenting incremental stimulus intensity (Faghihi and Moustafa, 2015).

It is critical to measure the efficiency of a single neuron and neural population to transfer information regarding different parameters involved in biological neural systems. Importantly, information theory has helped neuroscientists by proposing some measures of system efficiency such as mutual information (MI) (Deco and Schürmann, 1998; Mitaim and Kosko, 2004). MI quantifies the reduction of the uncertainty of a variable when we have knowledge of another variable. To quantify the neural system efficiency, MI between a single neuron and its neighbor has been measured (Roudi et al., 2009; Rowan, 2012). However, these studies have not considered connectivity rate and firing threshold as parameters involved in information processing by a single neuron. Moreover, MI was used to study the role of basic structural and functional parameters in information processing by neural population in insects’ olfactory systems (Faghihi et al., 2013). This study assigns an optimal value for feedback inhibition to encode different levels of odor concentration assuming that stimulus intensity affects just neurons’ firing rate. Importantly, stimuli information may be encoded by both changes in firing rate of single neurons and number of activated neurons in a neural ensemble.

**FIGURE 1:**
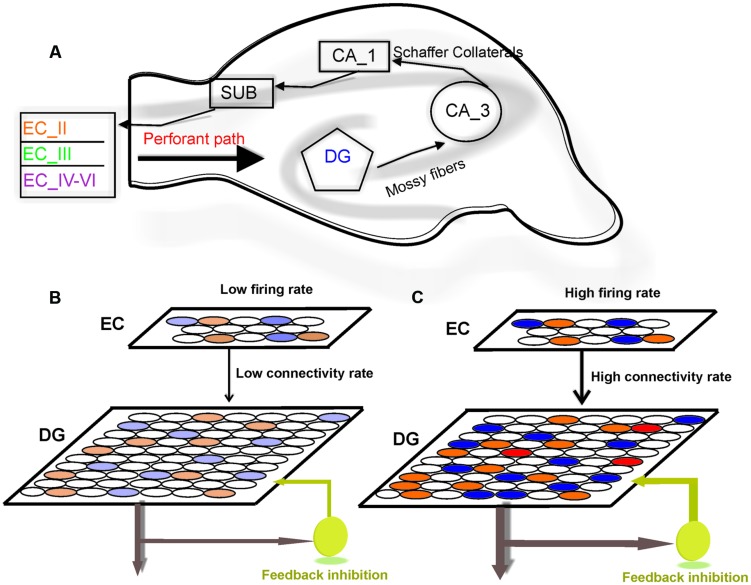
**Model structure**. **(A)** Information flow in the hippocampus. The perforant path is the major input to the hippocampus. The axons of the perforant path mainly arise in layer II of the entorhinal cortex (ECII). Axons from ECII/IV project to the granule cells of the DG. The mossy fibers are the axons of the DG granule cells and extend from the DG to CA3 pyramidal cells, forming their major input. Information is transferred by axons that project from the CA3 to the CA1 region. The information from CA1 to the subiculum (SUB) and on the entorhinal cortex (EC) performs the principal output from the hippocampus. **(B)** The model is composed of a neural network with 800 neurons in EC and 4000 neurons in the DG. Fully separated input pattern in EC may trigger separated neurons in the DG. **(C)** Increase in the number of activated neurons in EC or the connectivity rate between layers may lead to overlap in pattern of activated neurons in the DG (shown by red) which results in a decrease in pattern separation efficiency of the DG.

The hippocampus is a brain structure that plays a critical role in consolidating information from short-term memory into long-term memory. In the classic tri-synaptic pathway, information proceeds from the entorhinal cortex (EC) to the dentate gyrus (DG) to CA3 and then to CA1 which is known as the main hippocampal output (Van Strien et al., 2009; Newman and Hasselmo, 2014; **Figure 1A**). The animal’s brain ability to discriminate between similar experiences is a crucial feature of episodic memory. It is believed that information processing in the hippocampus complies with ‘compressed sensing theory’ (Petrantonakis and Poirazi, 2014). The formation of discrete representations in memory is thought to depend on a pattern separation process whereby cortical inputs are decorrelated as they enter the early stages of the hippocampus (Gilbert et al., 2001; Leutgeb et al., 2007). Computational models suggest that such function is dependent on pattern separation (Bakker et al., 2008; Yassa and Stark, 2011). Pattern separation is defined as the ability to transform a set of similar input patterns into a less-similar set of output patterns, which is believed to be dynamically regulated by hilar neurons. The storage capacity of such memory system, in terms of the number of patterns that can be stored and retrieved, is maximized if the patterns to be stored do not overlap extensively (Marr, 1970). In this context, overlap between patterns is defined as the degree to which individual elements in one pattern are also active in another pattern.

It is believed that the DG is involved in learning and memory (Gould et al., 1999). The DG is thought to contribute to spatial or episodic memory by functioning as a pattern separator (Leutgeb et al., 2007; Bakker et al., 2008). Given the premise that CA3 performs pattern storage and pattern completion, and that pattern separation can optimize CA3 function, the DG, which is a primary way station for entorhinal inputs traveling to CA3 was considered as pattern separation region in the hippocampus (Yeckel and Berger, 1990; Derrick, 2007). Pattern separation as a feature of the DG is performed by the low contact probability of dentate granule cell axons to CA3 pyramidal cells which could decrease the probability that two separate entorhinal input patterns activate the same subset of CA3 neurons (Myers and Scharfman, 2009). Moreover, to support this hypothesis, it has been observed that lesions of the DG circuitry result in impaired pattern separation-dependent memory (Gilbert et al., 1998). Pattern dysfunction impairment due to DG dysfunction may lead to declarative memory deficits in schizophrenia (Das et al., 2014).

The number of granule cells in the DG is approximately five times larger than the number of entorhinal cells (Amaral et al., 1990) where input pattern separation takes place (Bakker et al., 2008). The pattern separation process may be impaired in aging when the connectivity of these layers is affected (Spalding et al., 2013). For example, when a new event occurs, a sparse subset of neurons in the DG will respond. Studies examining the DG activity have found sparse coding from 2 to 4% of granule cells in a single context (Schmidt et al., 2012). Moreover, granule cells show a low firing rate compared with other brain regions (sparse spiking; Leutgeb et al., 2007; Piatti et al., 2013) which seems to be governed by GABAergic inhibition (Nitz and McNaughton, 2004). Information collected in the *Hippocampome* project (see: /hippocampome.org) about the location of soma, axon and dendrite of excitatory and inhibitory neuron types in the DG support the hypothesis of feedback inhibition as the main cause of sparse spiking of granule cells of the rodents’ hippocampus. In this work, we present a computational model of single neuron computation and separation efficiency of neural populations in the DG. The connectivity of single neuron to its neighbors in a neural population, firing threshold of neuron, impact of stimulus intensity and feedback inhibition are being considered as fundamental parameters involved in pattern separation in the DG. For this purpose, MI between inputs to a single neuron and its output is measured for different parameter values and different stimulus intensities.

The efficiency of neural systems to encode a stimulus is encoded by population of neurons. Therefore, a measure for pattern separation efficiency by the DG is presented. The model aims to describe optimal conditions which lead to a high pattern separation efficiency in the DG. This study additionally attempts to shed light on a theory of the role of impaired functional activity of inhibitory neurons in the DG and its implications in pattern separation deficiency observed in schizophrenia disorder.

## Materials and Methods

To model information processing in the DG, a probabilistic model of neural encoding efficiency of single neurons in the DG is presented to study the role of connectivity rate between the EC and the DG, firing probability of activated neuron in EC and feedback inhibition in efficiency of single neurons in the DG. Then a computational model of pattern separation efficiency of neural population of the DG is presented. The combination of these two calculations is being used to explain observed experimental data in the DG and pathological features of schizophrenia related to the DG dysfunction.

### Neural Encoding Efficiency of Single Neurons in the DG

To model information processing by single neurons we consider a single neuron in the DG and a neural layer composed of 100 neurons. The neural layer and the single neuron are connected according to a connectivity rate. The connectivity rate is defined as the probability of connection of single neurons in the DG to each neuron in the EC (one synapse between each neuron in the EC and a single neuron in the DG). For each connectivity rate, the connected neurons are randomly chosen from 100 neurons in the EC (**Figure 1B**). A stimulus is presented to the neural layer, which leads to the activation of a set of neuron in the neural layer. According to the connectivity vector between the single neurons in the DG neuron and the EC, the single neuron may receive some inputs. This number may increase if intensity of a stimulus is raised (**Figure 1C**). An inhibitory neuron may connect to the single neurons in the DG and can inhibit their activity as a function of firing probability of the single neuron.

To quantify the efficiency of the single neurons in the DG to encode its input, MI is measured between its output and inputs for different set of parameters values. The parameters that are involved in this calculation are:

S = 100: total number of neurons in neural layer
n = 0,…,S: number of connected neurons to single neuron
m = 0,…,S: number of activated neurons in neural layer by a stimulus
m′ = 0,…,S: number of inputs to single neuron (number of activated and connected neurons in neural layer)
r ∈ [0,1]: connectivity rate between neural layer and single neuron
p ∈ [0,1]: firing probability of neurons in neural layer
θ = 2,3,…,n: firing threshold of single neuron

Firing probability of the single neuron given *m* is calculated as Equation 1

$$P\left(T=1|m\right)=\Sigma_{m^{\prime }}^{m}{\;\;\Sigma }_{j=\theta }^{m^{\prime }}\left(\begin{array}{c} {\begin{array}{c} m \end{array}}^{\prime }\\ j \end{array}\right)p^{j}{\left(1-p\right)}^{m^{\prime }-j}\Sigma_{n=0}^{s}P\left(m^{\prime }|m,n,S\right)P\left(n|r,S\right)\,\;\;\;\left(1\right)$$

Where

*P* (n|r,S) is probability distribution of n

*P* (m′is probability distribution of m′

then

$\Sigma_{n=0}^{S}\,\;\;P\left(m^{\prime }|m,n,S\right)P\left(n|r,S\right)$

$\Sigma_{j=\theta }^{m^{\prime }}\,\;\;\left(\begin{array}{c} {\begin{array}{c} m \end{array}}^{\prime }\\ j \end{array}\right)p^{j}{\left(1-p\right)}^{m^{\prime }-j}\Sigma_{n=0}^{S}P\left(m^{\prime }|m,n,S\right)P\left(n|r,S\right)$ is firing probability of single neuron given m′

Mutual information is then calculated as Equation 2

$$MI=\Sigma_{m}P\left(T=1|m\right)\log\left(\frac{P\left(T=1|m\right)}{P(T)\;\;\;\;P\left(m\right)}\right)+\Sigma_{m}\;P(T=0|m)\log\,\;\left(\frac{P\left(T=0|m\right)}{P(T)\;\;\;P\left(m\right)}\right)\,\;\;\;\;\;\;\;\;\;\;\;\;\left(2\right)$$

Mutual information as efficiency measure of the single neuron is measured for different sets of structural and physiological parameters in the absence and presence of feedback inhibition. Moreover, feedback inhibition is involved in the system to observe its effect on MI. Feedback inhibition is modeled using Equation 3, which is modified from (Faghihi et al., 2013).

$$P_{I}=e^{-\alpha /f}\,\;\;\;\;\;\;\;\left(3\right)$$

Where P_I_ is the inhibition probability of each spike of the single neurons in the DG and f is average activity of its input. In the presence of feedback inhibition, MI (Equation 2) is calculated using modified firing probability of the single neuron (Equation 4).

$$P\left(T=1\right)=P\left(T=1\right)-e^{\frac{-\alpha }{P\left(T=1\right)}},if\,\;P\left(T=1\right)>e^{\frac{-\alpha }{P\left(T=1\right)}}\,\;\;\;\;\;\;\;\;\left(4\right)$$

Otherwise, *P* (*T*=1) = 0

### Pattern Separation Efficiency of Neural Populations in the Dentate Gyrus

The information of any event/stimulus which is presented to the hippocampus as a pattern of activated neurons in the ECII (briefly EC in the model) is transferred and encoded by activated neurons in the DG. The memory efficiency of the hippocampus is partially dependent on its capability to encode overlapped sets inputs from the EC as patterns of activated neurons in the DG with minimum overlap between patterns (Myers and Scharfman, 2009). The transferred information depends, on one the hand, on the connectivity rate between the EC and the DG, and on the other hand, on the firing rate of activated neurons in the EC. These two integrated parameters determine the population of activated neurons in the DG in response to a given input pattern. Eventually, each activated neuron has its own encoding efficiency (measured by MI) that is independent of other neurons or separation efficiency of the neural population in the DG. Hence, first separation efficiency of the DG is defined and measured in the simulation of a subset of real neuron numbers then a comprehensive explanation is presented for experimentally observed sparse coding and sparse spiking in the DG. The approximated number of neurons in many parts of rodent’s hippocampus is relatively known. The ratio of 1:5 is considered for number of neurons in the EC and the DG (actual numbers 200,000 and 1,000,000 for the EC and the DG, respectively). Keeping the ratio, we developed a neural network composed of two layers and an inhibitory neuron. The first layer and the second layer contained 800 and 4000 neurons, respectively (**Figure 1B,C**). The connectivity rate between the EC and the DG is unknown so it is assumed as a model parameter as values between zero and one which is defined as the probability of connecting each neuron in second layer to neurons in the first layer. The inhibitory neuron with different inhibition intensity is modeled as in (Faghihi and Moustafa, 2015). The activity of neurons in the first layer (the EC) is modeled as firing probability in each time bin. Totally, 500 time bins, each equal to 10 ms, were used to simulate neural activity of input pattern. The neural activity of neurons in the second layer (the DG) was modeled as integrate and fire model using electrophysiological features of granule cells in the rodent’s DG (Lübke et al., 1998). In this work, we have assumed that the input patterns from the EC are fully separable (overlap equal to zero). This assumption allows for studying the role of different structural and physiological parameter values on the DG efficiency to separate inputs. However, modeling transferred information from the DG into CA3 needs considering overlapped inputs pattern from the EC and how the pattern completion is performed in CA3 (Gold and Kesner, 2005).

For each pair of firing rate of activated neurons in the EC and connectivity rate between the EC and the DG, inputs as fully separated sets of activated neurons in the EC were presented to the neural network. In the simulations here, the number of activated neurons in an input pattern of the EC was equal to 20, so the number of input patterns from the EC in any simulation was equal to 40 (totally 800 neurons in the simulated EC). The activation patterns in the DG neurons were compared to calculate their degree of separation as ‘separation efficiency (*S*).’

The activation pattern in the DG is presented as columns of input patterns: [I_1,_…,I_L_] and rows of DG neurons [n_1_,…,n_k_].

$$S=\frac{\left(\Sigma_{i=1}^{K}\Sigma_{j=1}^{L}\left(1-\frac{N_{i,j}}{L}\right)\right)}{K}:\left[0,1\right]\,\;if\,\;\;N_{i,j}>0\,\;\;\;\;\left(5\right)$$

N_i,j_ ≤ L; K ≤ 4000

Where ‘K’ is the number of activated neurons in the DG in response to inputs presentation and ‘*N*’ is the number of activation of each neuron in the DG by ‘*L*’ as the number of separated sets of activated neurons in the EC (input patterns). *S* is based on the percentage of 40 inputs from the EC which activated a given neuron in the DG. Such quantity is calculated and summed up for all DG neurons. Minimizing this overlap, maximizing S as a measure of the DG separation efficiency. As there are large possibilities to choose sets of separable sets of neurons in the EC, we derived the average of *S* by measuring 500 trials (each trial as 40 sets of 20 fully separated neurons in the EC) for each pair of connectivity rate between the EC and the DG, and firing probability of activated neurons in the EC. Feedback inhibition with different parameters values are used to study the role of inhibition in separation efficiency of the DG.

## Results

**FIGURE 2:**
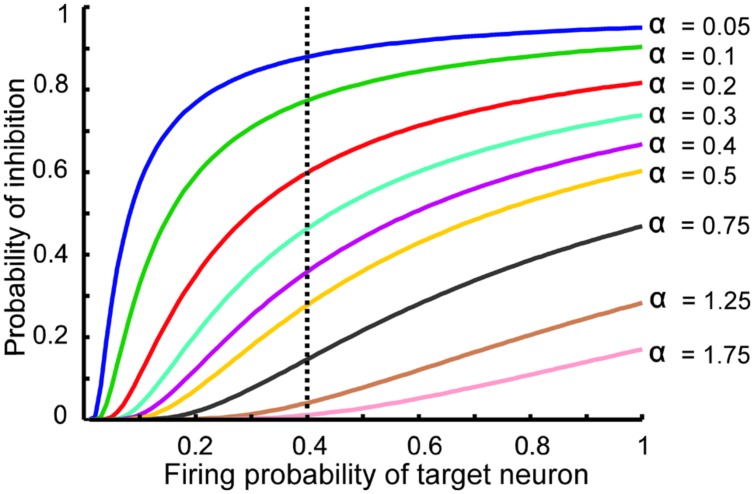
**The relationship between the probability of feedback inhibition and firing probability of the single neurons**. For different parameter value (α), average firing probability of the single neurons elicits activity of inhibitory neuron. The vertical dash line shows the role of inhibition parameter for different inhibition intensity for average firing probability of single neurons equal to 0.4.

The information processing in the DG is performed by single neurons that receive information from the EC activated neurons. This process depends on its connectivity to other neurons and firing probability of activated and connected neurons. Therefore, to quantify efficiency of a single neuron to encode its input, the MI between output of the single neuron and its input for different connectivity rates and firing probability of inputs is measured. Feedback inhibition may play important role in information processing in the hippocampus. **Figure 2** shows the relationship between ‘input intensity’ into inhibitory neuron and ‘inhibition intensity’ into the single neuron.

**FIGURE 3:**
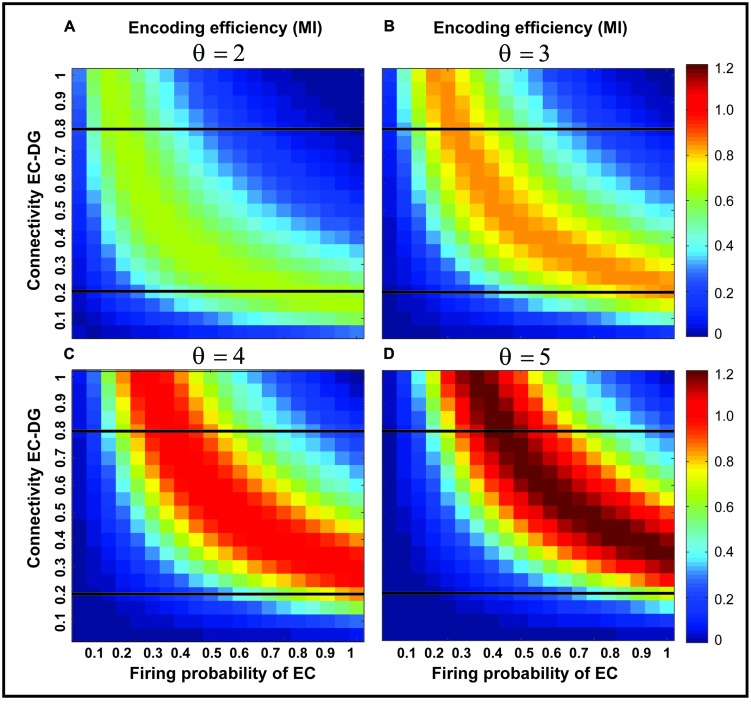
**Mutual information for different parameters values**. Mutual information is calculated for a connectivity rate between the EC and the single neuron, firing probability of activated neurons in the neural layer and firing threshold of the single neuron between 2 and 5 **(A–D)**. An increase in firing threshold leads to a shift of high mutual information values to right (higher firing probability of neurons in the neural layer) for all connectivity rates. Two connectivity rates (0.2 and 0.8) are studied (shown in **Figure 4**).

**FIGURE 4:**
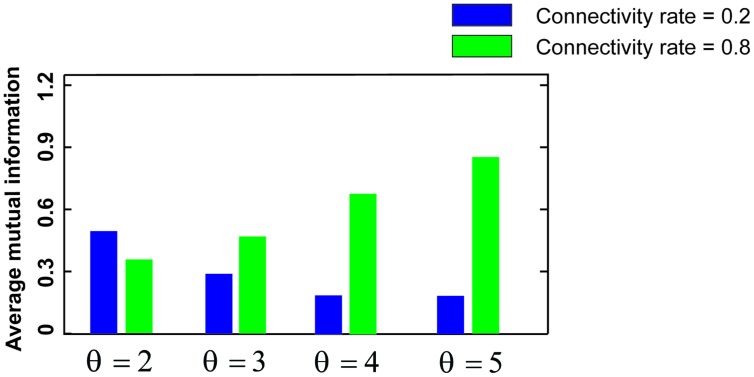
**Mutual information for low and high connectivity rates**. The average mutual information for a low connectivity rate equal to 0.2 is decreased by increasing firing threshold while it is increased for high connectivity rate equal to 0.8.

**Figure 3** shows the MI for different firing thresholds of the single neuron. The results illustrate a non-linear dependency of MI on connectivity rate and firing probability of inputs. An increase in firing threshold leads to a shift of high MI to right. **Figure 4** shows MI for different firing threshold at low and high connectivity rate, 0.2 and 0.8, respectively. The results show that for a low connectivity rate (equal to 0.2) increase in θ leads to a decrease in average MI while it increases MI for high connectivity rate.

**FIGURE 5:**
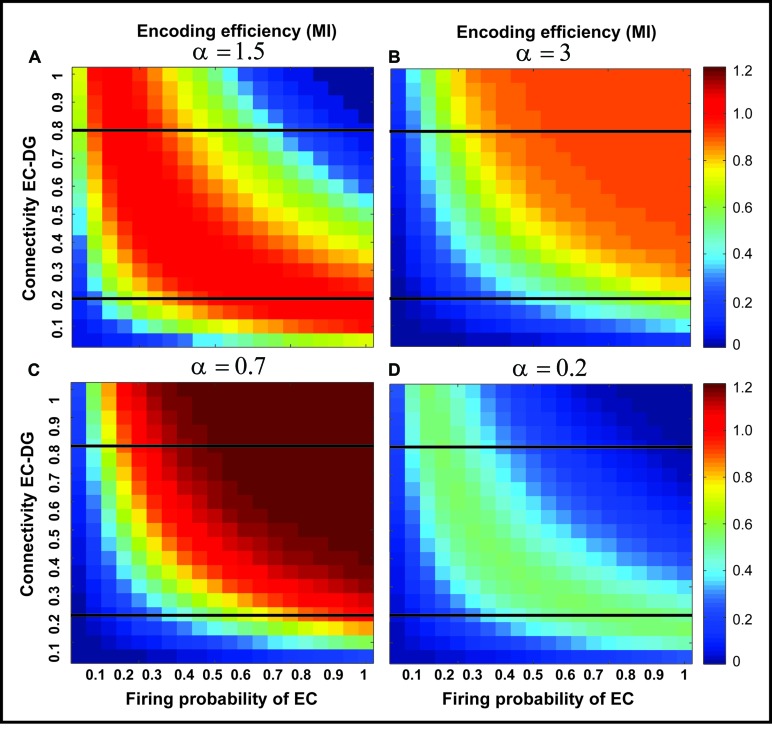
**The effect of feedback inhibition on mutual information**. Mutual information as single neuron’s encoding efficiency for all connectivity rates and firing probability depends on the feedback inhibition parameter value. A decrease in inhibition parameter value leads to an increase in mutual information **(A–C)** but lower values (high inhibition intensity) lead to a decrease in mutual information **(D)**. This observation motivates the search for an optimal inhibition parameter.

To generalize the simulation of the effect of feedback inhibition on MI of a single neuron, MI was measured for different inhibition parameter values (**Figure 5**). The results show the existence of a possible optimal inhibition parameter value for a given connectivity rate (see also **Figure 2**).

**FIGURE 6:**
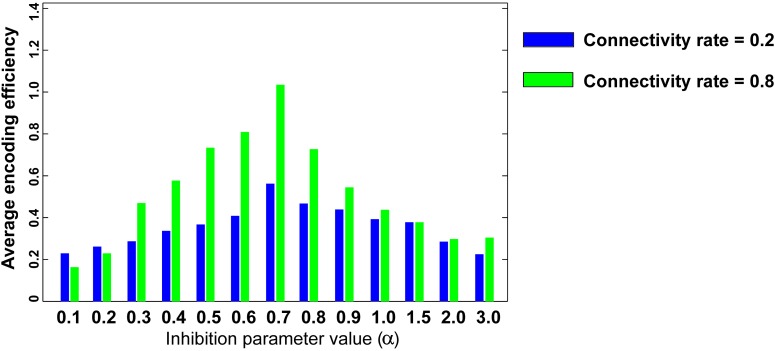
**Optimal inhibition parameter for low and high connectivity rates**. Average mutual information (single neuron’s encoding efficiency) was measured for θ =2 and for connectivity rates equal to 0.2 and 0.8. The results show that optimal inhibition parameter (α) for both connectivity rates is 0.7.

**Figure 6** shows the optimal inhibition parameter equal to 0.7 for both low and high connectivity rates.

**FIGURE 7:**
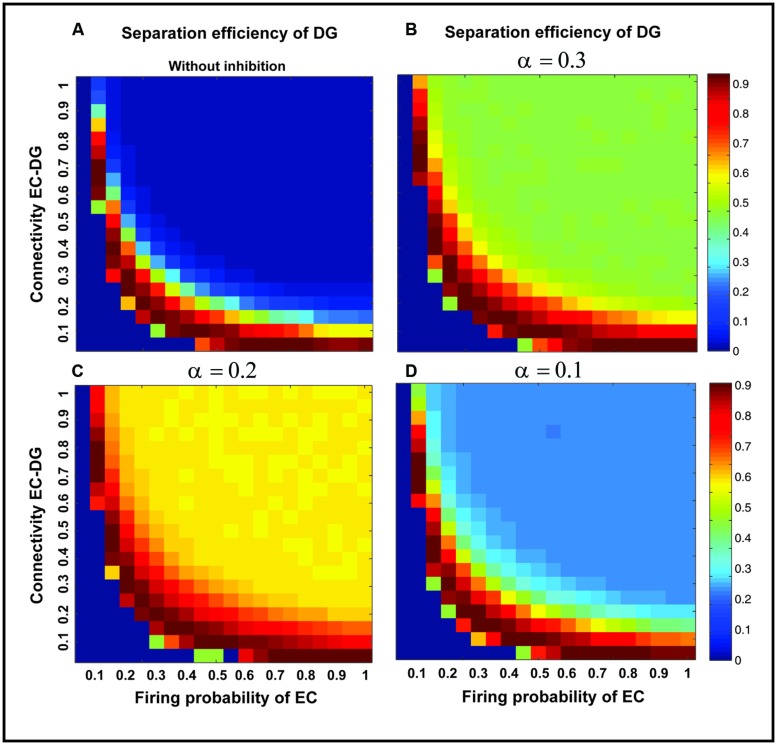
**Separation efficiency of the DG for different firing probabilities of activated neurons in the EC, and connectivity rates between the EC and DG**. **(A)** In the absence of feedback inhibition, an increase in connectivity rate leads to a decrease in separation efficiency when incremental firing probability of neurons in the EC is presented. **(B)** In the presence of feedback inhibition, different inhibition intensities as different α values leads to different separation efficiency (α =0.1). **(C)** α =0.2 **(D)** α =0.3. Feedback inhibition with α =0.2 leads to high separation efficiency while decrease in α value (high inhibition intensity) causes deficiency in separation of pattern by suppressing the activity of neurons in the DG.

**FIGURE 8:**
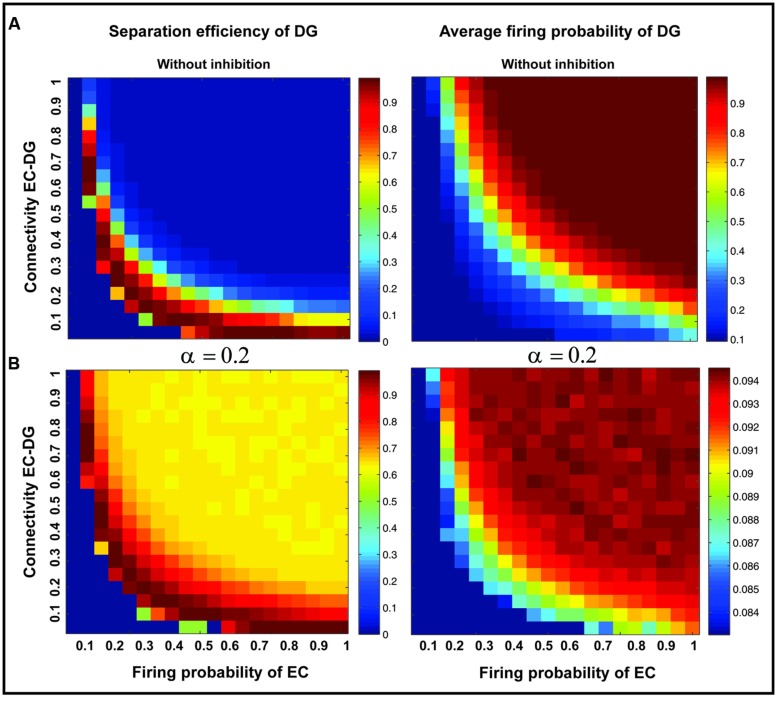
**Separation efficiency of the DG versus average firing probability of neurons in the DG**. **(A)** In absence of feedback inhibition separation efficiency is decreased by increase in firing probability of neurons in the EC for connectivity rates higher than 0.1 (left panel) while the average firing probability of activated neurons in the DG is raised (right panel). **(B)** In presence of optimal inhibition parameter (α =0.2) increase in separation efficiency for all connectivity rates and firing probability of inputs in the EC is observed (left panel) while the average firing probability of activated neurons in the DG is decreased remarkably as a consequence of high inhibition intensity (right panel).

**Figure 7** shows the separation efficiency of the DG calculated for different firing probabilities of activated neurons in the EC and connectivity rate between the EC and the DG. **Figure 7A** shows the separation efficiency in the absence of feedback inhibition effect on neural activity of the DG. For low connectivity rate (between 0.05 and 0.15), an increase in firing probability leads to an increase in separation efficiency while for higher connectivity rates it causes decrease in separation efficiency. The cause of a decrease in separation efficiency for higher firing rates is the increase in the number of activated neurons in the DG in response to separated inputs (activated sets of neurons in the EC) which consequently results in the overlap of the DG activated neurons by separated inputs from the EC. Moreover, increase in the number of activated neurons in the DG associates with an increase in the average firing frequency of activated neurons in the DG (**Figure 8A** right panel). Feedback inhibition with a different α value leads to change in separation efficiency for different connectivity rates of the EC and DG, and firing probability of the EC. The optimal parameter value to obtain maximum separation efficiency among the α values (0.05, 0.1, 0.15,...1) is α =0.2 (**Figure 7A–D**). The optimal α value (to obtain maximum pattern separation efficiency) causes a low average firing frequency in activated neurons in the DG in response to inputs from the EC (**Figure 8** right panel). To obtain maximum separation efficiency, feedback inhibition with high inhibition intensity (α =0.2) is required when different probabilities of inputs are presented to the DG.

**FIGURE 9:**
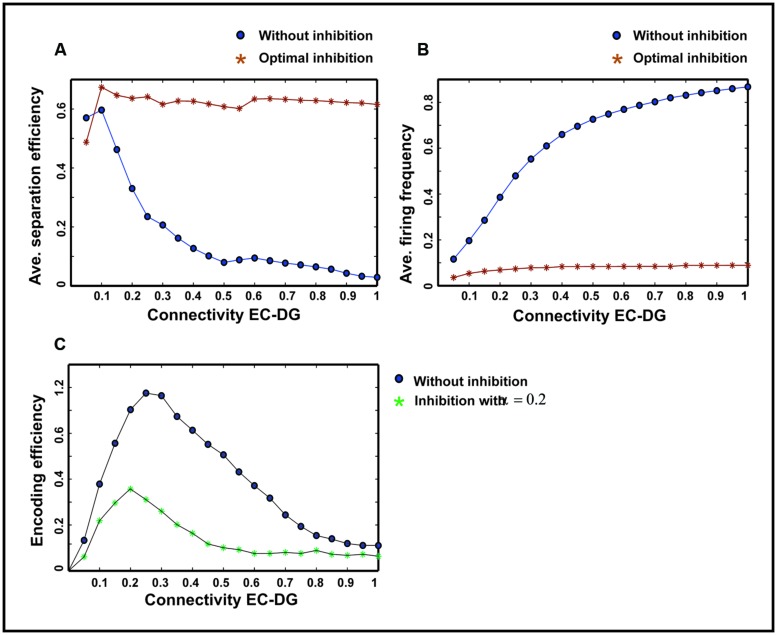
**Comparing average separation efficiency of the DG, the average firing frequency of the DG and average single neurons efficiency in the DG for optimal inhibition parameter value and without inhibition**. **(A)** Average separation efficiency over different firing probability of EC. In the presence of optimal inhibition for all connectivity rates between the EC and DG, high separation efficiency for the DG is obtained while in the absence of inhibition increase in connectivity leads to low separation efficiency. **(B)** Average firing frequency over different firing probability of EC. In the presence of optimal inhibition parameter value very low firing frequency is obtained for all connectivity rates between the EC and the DG. **(C)** Average single neurons encoding efficiency over different firing probability of EC. In the presence of optimal inhibition parameter value, low MI as the measure of encoding efficiency is obtained, comparing to the absence of inhibition for different connectivity rates between the EC and the DG.

The input to the EC may have different intensities as different firing probability of neurons in the EC. Therefore, the average pattern separation efficiency for different connectivity between the EC and the DG was measured over firing probabilities of the EC (**Figure 9A**). The results show that α = 0.2 helps the DG to keep its average separation efficiency at high level (optimal inhibition parameter in regard to maximum separation efficiency; **Figure 9A**). This inhibition parameter value causes a low firing frequency in activated neurons in the DG (**Figure 9B**). The inhibition intensity equal to 0.2 (α = 0.2) leads to a low encoding efficiency as compared to the absence of inhibition (**Figure 9C**).

## Discussion

Animals receive large amounts of information from different source of stimuli and encode them efficiently in different parts of their brains. Neural encoding is essentially performed by single neurons while the functionality of neural systems strongly depends on the activity of neural populations. Single neurons in a neural ensemble integrate their inputs and generate output as spiking trains with different firing rates. Synchronization of neural activities and their role in neural systems is an example of population coding performed by the animal brain (Jiao et al., 2015). Therefore, encoding by single neurons and neural populations is critical to study the role of structural and functional parameters involved in information processing. Moreover, finding these parameter values shed light on the principles of designing efficient bio-inspired neural systems. Years of research have demonstrated the vital role of the hippocampus in information processing including memory. Although many neuron types in rodent’s hippocampus have been detected and named, however, the role of inhibitory neurons and their high variability is not known. Specially, the balanced excitation and inhibition plays a critical role in neural communication (Roux and Buzsáki, 2014; Vega-Flores et al., 2014). In addition, the connectivity rate of different regions of hippocampus is under research as it may play an important role in normal and disorders with hippocampal dysfunction (e.g., Schizophrenia). The main information pathway in hippocampus is known such that separation of input patterns presented to the ECII has been assigned to the DG (Yassa and Stark, 2011). Hence, theoretical studies and simulations may help to study structural and functional parameters in the hippocampus that are difficult to assess by experiments. Pattern separation as a critical function of the DG depends on many parameters that we have modeled in the current study. Two well-known phenomena in the DG are ‘sparse coding’ as low number of activated neurons in the DG (about 4%) and ‘sparse spiking of single neurons.’ Sparse coding which may play a fundamental role in pattern separation capability of the DG. However, the role of sparse spiking of the DG excitatory neurons (low firing rate of DG neurons) in information processing of the DG has not been studied yet. It is noticeable to consider the role of neurons number in the EC and the DG with ratio about 1:5 which may act as an important structural feature of hippocampus to diverge information from the EC to the DG and so consequently to improve pattern separation efficiency of the DG. Such divergences of information and sparse spiking have been detected in the Mushroom Body as insects’ memory center (Heisenberg, 2003). Therefore, sparse coding and sparse spiking may be common features of animals’ memory centers which enable them to store huge amount of information with different intensities.

In this work, we developed a computational model using experimental information available on hippocampus to address some questions. The first problem is the role of connectivity rate between the EC and the DG, and firing probability of activated neurons in the EC in the separation efficiency of the DG. The next question is about information transferred from the EC into the DG neurons regarding their low firing frequency. The last problem is to study the role of feedback inhibition in pattern separation efficiency of the DG and sparse spiking of the DG neurons.

Modeling of a single neuron’s encoding efficiency has shown that high or low connectivity rate of the EC and the DG and/or low or high firing probability of activated neurons in the EC may cause remarkable decrease in encoding efficiency of single neurons in the DG. It occurs as a consequence of low variation in spiking pattern of neurons (vanSteveninck et al., 1997). In this process, feedback inhibition with optimal intensity (here as 0.7) can play an important role in neural encoding of single neurons for different connectivity rates of the EC and the DG, and firing probability of neurons in the EC. Different connectivity rates of the EC and the DG, and firing probability of the EC neurons affect the number of activated neurons in neural population of the DG and their firing probabilities as well. Therefore, one can expect that there are optimal values for these parameters to obtain maximum separation efficiency of the DG. In this study, simulations have shown that in the absence of feedback inhibition, for low connectivity rate between the EC and the DG (<0.15) for all firing probabilities of the EC neurons, high separation efficiency is obtained. For other connectivity values, increase in firing probability of the EC neurons can lead to decrease in separation efficiency. Further, feedback inhibition with an optimal value (about 0.2) can help the DG to illustrate high separation efficiency when incremental firing probability of the EC neurons is presented. However, this optimal inhibition intensity which results in high separation efficiency, on one the hand, leads to low encoding efficiency, and on the other hand, causes low firing frequency of the DG neurons (on average 0.06).

These simulations explain why neurons in the DG have low firing frequency (sparse spiking) and high separation efficiency. Low firing frequency of the DG neurons may be considered as the expense of having high separation efficiency but low encoding efficiency of single neurons may lead to the deficiency of the DG to encode small changes in input’s intensity. In other words, similar input patterns should not be encoded in the DG. The high capability of the DG to separate similar patterns regarding its very low single neuron’s encoding efficiency may be done by the activation of very low number of neurons in the DG. Experiments have shown that about 4% of the DG neurons are being activated in response to input presentation to the EC. Therefore, the DG is able to separate very similar input patterns to encode small fluctuations in input intensity. In this study, we assume that inputs have no overlap and it was used to measure separation of fully separated inputs for different structural and physiological parameters. For future works, the separation efficiency of the DG should be measured for inputs with different levels of similarities (overlapped inputs from the EC) to model information processing from the DG to CA3 and CA1. The simulations assign a critical role for feedback inhibition on the DG neurons by interneurons with an optimal parameter value that leads to low single neurons efficiency. The neural substrate of the parameter value which was modeled as the inhibition intensity by interneurons can be considered as the amount of neurotransmitter release like GABA in response to the sum of inputs to the interneuron. For better understanding of inhibitory neurons, it is required to model interneurons’ activity using electrophysiological data and neural activity models. Such improvement helps to explore the role of inhibitory synaptic plasticity and its role in disorders related to the DG dysfunctions (Flores and Méndez, 2014; Griffen and Maffei, 2014; Wang and Maffei, 2014).

Schizophrenia patients often show functional abnormalities in memory. The dysfunction in the DG may lead to impairments in memory and cognition capability. Moreover, molecular and cellular evidences motivate to consider the DG as a brain region involved in Schizophrenia (Das et al., 2014). The impaired functionality of the DG to CA3 may reduce the pattern separation efficiency (Tamminga et al., 2010). Furthermore, reduction in number of inhibitory interneurons in hippocampus has been explored in Schizophrenia (Konradi et al., 2011). The experimental information integrated with the model presented here propose a critical role of feedback inhibition in information processing in the DG as pattern separator and its impairment in Schizophrenia; the model predicts impaired inhibitory activity in the DG as a cause of the disorder which should be experimentally checked.

The computational model presented in this work illustrates the importance of the combination of theoretical measures with simulations to understand the role of structural and physiological parameters in biological neural systems. It also allows to model information processing in any kind of neural system independent of specific neural architectures or physiological constraints. The model proposes a system neuroscience perspective on the architecture of neural systems with high efficiency of information processing independent of physiological constraints. Future work should include other parameters or mechanisms that have been discovered in neural communication to check their impact on information processing by single neurons and neural populations as well. Some of these mechanisms include synaptic computation and unreliable synapses where spiking dose not results in neurotransmitter release.

## References

[B1] Amaral D. G. Ishizuka N. Claiborne B. (1990). Chapter neurons, numbers and the hippocampal network. *Prog. Brain Res.* 83 1–11 10.1016/S0079-6123(08)61237-6 2203093

[B2] Bakker A. Kirwan C. B. Miller M. Stark C. E. (2008). Pattern separation in the human hippocampal CA3 and dentate gyrus. *Science* 319 1640–1642 10.1126/science.1152882 18356518 PMC2829853

[B3] Barres B. A. Silverstein B. E. Corey D. P. Chun L. L. (1988). Immunological, morphological, and electrophysiological variation among retinal ganglion cells purified by panning. *Neuron* 1 791–803 10.1016/0896-6273(88)90127-4 2908449

[B4] Clayton N. S. Hen R. (2005). Neural circuits and behaviour: developmental and evolutionary perspectives. *Curr. Opin. Neurobiol.* 15 683–685 10.1016/j.conb.2005.10.007 16271456

[B5] D’Amelio M. Rossini P. M. (2012). Brain excitability and connectivity of neuronal assemblies in Alzheimer’s disease: from animal models to human findings. *Prog. Neurobiol.* 99 42–60 10.1016/j.pneurobio.2012.07.001 22789698

[B6] Das T. Ivleva E. I. Wagner A. D. Stark C. E. Tamminga C. A. (2014). Loss of pattern separation performance in schizophrenia suggests dentate gyrus dysfunction. *Schizophr. Res.* 159 193–197 10.1016/j.schres.2014.05.006 25176349 PMC4177293

[B7] Deco G. Schürmann B. (1998). Stochastic resonance in the mutual information between input and output spike trains of noisy central neurons. *Physica D Nonlinear Phenomena* 117 276–282 10.1016/S0167-2789(97)00313-8

[B8] Derrick B. E. (2007). Plastic processes in the dentate gyrus: a computational perspective. *Prog. Brain Res.* 163 417–451 10.1016/S0079-6123(07)63024-6 17765732

[B9] Ebner M. Hameroff S. (2011). Lateral information processing by spiking neurons: a theoretical model of the neural correlate of consciousness. *Comput. Intell. Neurosci.* 2011:11 10.1155/2011/247879 PMC3199212 22046178

[B10] English D. F. Peyrache A. Stark E. Roux L. Vallentin D. Long M. A. (2014). Excitation and inhibition compete to control spiking during hippocampal ripples: intracellular study in behaving mice. *J. Neurosci.* 34 16509–16517 10.1523/JNEUROSCI.2600-14.2014 25471587 PMC4252557

[B11] Faghihi F. Kolodziejski C. Fiala A. Wörgötter F. Tetzlaff C. (2013). An information theoretic model of information processing in the *Drosophila* olfactory system: the role of inhibitory neurons for system efficiency. *Front. Comput. Neurosci.* 7:183 10.3389/fncom.2013.00183 PMC3868887 24391579

[B12] Faghihi F. Moustafa A. A. (2015). Impaired homeostatic regulation of feedback inhibition associated with system deficiency to detect fluctuation in stimulus intensity: a simulation study. *Neurocomputing* 151 1248–1254 10.1016/j.neucom.2014.11.008

[B13] Flores C. E. Méndez P. (2014). Shaping inhibition: activity dependent structural plasticity of GABAergic synapses. *Front. Cell. Neurosci.* 8:327 10.3389/fncel.2014.00327 PMC4209871 25386117

[B14] Gilbert P. E. Kesner R. P. DeCoteau W. E. (1998). Memory for spatial location: role of the hippocampus in mediating spatial pattern separation. *J. Neurosci.* 18 804–810. 9425021 10.1523/JNEUROSCI.18-02-00804.1998 PMC6792543

[B15] Gilbert P. E. Kesner R. P. Lee I. (2001). Dissociating hippocampal subregions: a double dissociation between dentate gyrus and CA1. *Hippocampus* 11 626–636 10.1002/hipo.1077 11811656

[B16] Gold A. E. Kesner R. P. (2005). The role of the CA3 subregion of the dorsal hippocampus in spatial pattern completion in the rat. *Hippocampus* 15 808–814 10.1002/hipo.20103 16010664

[B17] Gould E. Tanapat P. Hastings N. B. Shors T. J. (1999). Neurogenesis in adulthood: a possible role in learning. *Trends Cogn. Sci.* 3 186–192 10.1016/S1364-6613(99)01310-8 10322475

[B18] Griffen T. C. Maffei A. (2014). GABAergic synapses: their plasticity and role in sensory cortex. *Front. Cell. Neurosci.* 8:91 10.3389/fncel.2014.00091 PMC3972456 24723851

[B19] Gruart A. Sánchez-Campusano R. Fernández-Guizán A. Delgado-García J. M. (2014). A differential and timed contribution of identified hippocampal synapses to associative learning in mice. *Cereb. Cortex* 10.1093/cercor/bhu054 [Epub ahead of print]. 24654258

[B20] Gupta A. Vig L. Noelle D. C. (2012). A neurocomputational approach to automaticity in motor skill learning. *Biol. Inspired Cogn. Archit.* 2 1–12 10.1016/j.bica.2012.07.009

[B21] He Y. Chen Z. Gong G. Evans A. (2009). Neuronal networks in Alzheimer’s disease. *Neuroscientist* 15 333–350 10.1177/1073858409334423 19458383

[B22] Heisenberg M. (2003). Mushroom body memoir: from maps to models. *Nat. Rev. Neurosci.* 4 266–275 10.1038/nrn1074 12671643

[B23] Jiao X. Zhu D. Wang R. (2015). “Synchronization in neuronal population with phase response,” in *Advances in Cognitive Neurodynamics (IV),* ed. Liljenström H. (Dordrecht: Springer), 259–263.

[B24] Konradi C. Yang C. K. Zimmerman E. I. Lohmann K. M. Gresch P. Pantazopoulos H. (2011). Hippocampal interneurons are abnormal in schizophrenia. *Schizophr. Res.* 131 165–173 10.1016/j.schres.2011.06.007 21745723 PMC3159834

[B25] Lamprecht R. LeDoux J. (2004). Structural plasticity and memory. *Nat. Rev. Neurosci.* 5 45–54 10.1038/nrn1301 14708003

[B26] Leutgeb J. K. Leutgeb S. Moser M. B. Moser E. I. (2007). Pattern separation in the dentate gyrus and CA3 of the hippocampus. *Science* 315 961–966 10.1126/science.1135801 17303747

[B27] Lübke J. Frotscher M. Spruston N. (1998). Specialized electrophysiological properties of anatomically identified neurons in the hilar region of the rat fascia dentata. *J. Neurophysiol.* 79 1518–1534. 9497429 10.1152/jn.1998.79.3.1518

[B28] Marjovi A. Marques L. (2011). Multi-robot olfactory search in structured environments. *Rob. Auton. Syst.* 59 867–881 10.1016/j.robot.2011.07.010

[B29] Marr D. (1970). A theory for cerebral neocortex. *Pro. R. Soc. Lond. B Biol. Sci.* 176 161–234 10.1098/rspb.1970.0040 4394740

[B30] Mitaim S. Kosko B. (2004). Adaptive stochastic resonance in noisy neurons based on mutual information. *IEEE Trans. Neural Netw.* 15 1526–1540 10.1109/TNN.2004.826218 15565779

[B31] Myers C. E. Scharfman H. E. (2009). A role for hilar cells in pattern separation in the dentate gyrus: a computational approach. *Hippocampus* 19 321–337 10.1002/hipo.20516 18958849 PMC2723776

[B32] Newman E. L. Hasselmo M. E. (2014). CA3 Sees the big picture while dentate gyrus splits hairs. *Neuron* 81 226–228 10.1016/j.neuron.2014.01.004 24462091

[B33] Nitz D. McNaughton B. (2004). Differential modulation of CA1 and dentate gyrus interneurons during exploration of novel environments. *J. Neurophysiol.* 91 863–872 10.1152/jn.00614.2003 14523073

[B34] Petrantonakis P. C. Poirazi P. (2014). A compressed sensing perspective of hippocampal function. *Front. Syst. Neurosci.* 8:141 10.3389/fnsys.2014.00141 PMC4126371 25152718

[B35] Piatti V. C. Ewell L. A. Leutgeb J. K. (2013). Neurogenesis in the dentate gyrus: carrying the message or dictating the tone. *Front. Neurosci.* 7:50 10.3389/fnins.2013.00050 PMC3616253 23576950

[B36] Roudi Y. Nirenberg S. Latham P. E. (2009). Pairwise maximum entropy models for studying large biological systems: when they can work and when they can’t. *PLoS Comput. Biol.* 5:e1000380 10.1371/journal.pcbi.1000380 PMC2674569 19424487

[B37] Roux L. Buzsáki G. (2014). Tasks for inhibitory interneurons in intact brain circuits. *Neuropharmacology* 30:e14 10.1016/j.neuropharm.2014.09.011 PMC4254329 25239808

[B38] Rowan M. (2012). Information-selectivity of beta-amyloid pathology in an associative memory model. *Front. Comput. Neurosci.* 6:2 10.3389/fncom.2012.00002 PMC3260488 22279434

[B39] Rozo L. Jiménez P. Torras C. (2013). A robot learning from demonstration framework to perform force-based manipulation tasks. *Intel. Serv. Robotics* 6 33–51 10.1007/s11370-012-0128-9

[B40] Schmidt B. Marrone D. F. Markus E. J. (2012). Disambiguating the similar: the dentate gyrus and pattern separation. *Brain Res.* 226 56–65 10.1016/j.bbr.2011.08.039 21907247

[B41] Sengupta B. Laughlin S. B. Niven J. E. (2013). Balanced excitatory and inhibitory synaptic currents promote efficient coding and metabolic efficiency. *PLoS Comput. Biol.* 9:e1003263 10.1371/journal.pcbi.1003263 PMC3789774 24098105

[B42] Seung H. S. Sümbül U. (2014). Neuronal cell types and connectivity: lessons from the retina. *Neuron* 83 1262–1272 10.1016/j.neuron.2014.08.054 25233310 PMC4206525

[B43] Song K. Liu Q. Wang Q. (2011). Olfaction and hearing based mobile robot navigation for odor/sound source search. *Sensors* 11 2129–2154 10.3390/s110202129 22319401 PMC3274022

[B44] Spalding K. L. Bergmann O. Alkass K. Bernard S. Salehpour M. Huttner H. B. (2013). Dynamics of hippocampal neurogenesis in adult humans. *Cell* 153 1219–1227 10.1016/j.cell.2013.05.002 23746839 PMC4394608

[B45] Stramandinoli F. Marocco D. Cangelosi A. (2012). The grounding of higher order concepts in action and language: a cognitive robotics model. *Neural Netw.* 32 165–173 10.1016/j.neunet.2012.02.012 22386502

[B46] Tamminga C. A. Stan A. D. Wagner A. D. (2010). The hippocampal formation in schizophrenia. *Am. J. Psychiatry* 167 1178–1193 10.1176/appi.ajp.2010.09081187 20810471

[B47] vanSteveninck R. R. D. R. Lewen G. D. Strong S. P. Koberle R. Bialek W. (1997). Reproducibility and variability in neural spike trains. *Science* 275 1805–1808 10.1126/science.275.5307.1805 9065407

[B48] Van Strien N. M. Cappaert N. L. M. Witter M. P. (2009). The anatomy of memory: an interactive overview of the parahippocampal–hippocampal network. *Nat. Rev. Neurosci.* 10 272–282 10.1038/nrn2614 19300446

[B49] Vega-Flores G. Gruart A. Delgado-García J. M. (2014). Involvement of the GABAergicsepto-hippocampal pathway in brain stimulation reward. *PLoS ONE* 9:e113787 10.1371/journal.pone.0113787 PMC4263242 25415445

[B50] Wang L. Maffei A. (2014). Inhibitory plasticity dictates the sign of plasticity at excitatory synapses. *J. Neurosci.* 34 1083–1093 10.1523/JNEUROSCI.4711-13.2014 24453301 PMC3898280

[B51] Wilbrecht L. Shohamy D. (2009). Neural circuits can bridge systems and cognitive neuroscience. *Front. Hum. Neurosci.* 3:81 10.3389/neuro.09.081.2009 PMC2814556 20126435

[B52] Yassa M. A. Stark C. E. (2011). Pattern separation in the hippocampus. *Trends Neurosci.* 34 515–525 10.1016/j.tins.2011.06.006 21788086 PMC3183227

[B53] Yeckel M. F. Berger T. W. (1990). Feedforward excitation of the hippocampus by afferents from the entorhinal cortex: redefinition of the role of the trisynaptic pathway. *Proc. Natl. Acad. Sci. U.S.A.* 87 5832–5836 10.1073/pnas.87.15.5832 2377621 PMC54422

[B54] Yue S. Rind F. C. Keil M. S. Cuadri J. Stafford R. (2006). A bio-inspired visual collision detection mechanism for cars: optimization of a model of a locust neuron to a novel environment. *Neurocomputing* 69 1591–1598 10.1016/j.neucom.2005.06.017

[B55] Zawadzki K. Feenders C. Viana M. P. Kaiser M. Costa L. D. F. (2012). Morphological homogeneity of neurons: searching for outlier neuronal cells. *Neuroinformatics* 10 379–389 10.1007/s12021-012-9150-5 22615032

